# Supplementary material for: Differences in Glycolysis and Mitochondrial Respiration between Cytotrophoblast and Syncytiotrophoblast In-Vitro: Evidence for Sexual Dimorphism
Source: Int J Mol Sci. 2021 Oct 8;22(19):10875. doi: 10.3390/ijms221910875 (PMC8509198; doi:10.3390/ijms221910875)

SUPPLEMENTARY FIGURES:

Supplemental Figure S1A: Quantification of beta hCG production in male and female CT vs ST

Data plotted as individual values of paired CT and ST from the same sample Male (blue, n=4) and female (pink, n=4). *p<0.05, **p<0.01, (paired t test, CT vs ST within same fetal sex group, and # p<0.05, (Mann-Whitney test, male vs female). **1B: Confriming syncytialization.** Representative images showing E-cadherin (red) surrounding multiple nuclei (blue) at 96hrs of culture under standard conditions outlined in the methods section.

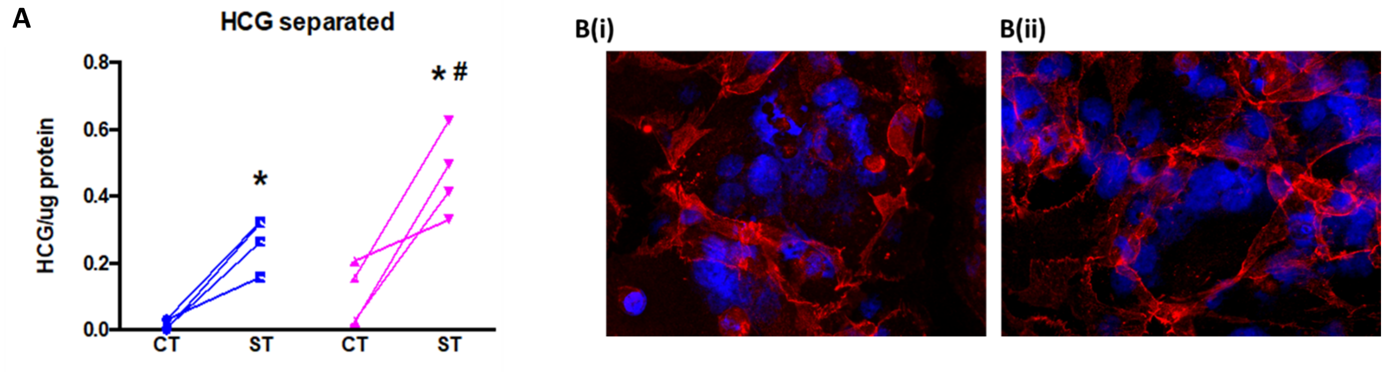

Supplemental Figure S2. Glycolytic function in male and female CT vs. ST

A) Non-glycolytic acidification, B) Glycolysis, C) Glycolytic capacity and D) Glycolytic reserve separated by fetal sex. Male (blue, n=8) and female (pink, n=8). Data plotted as individual values of paired CT and ST from the same sample. *P<0.05 (Wilcoxon test CT vs ST).

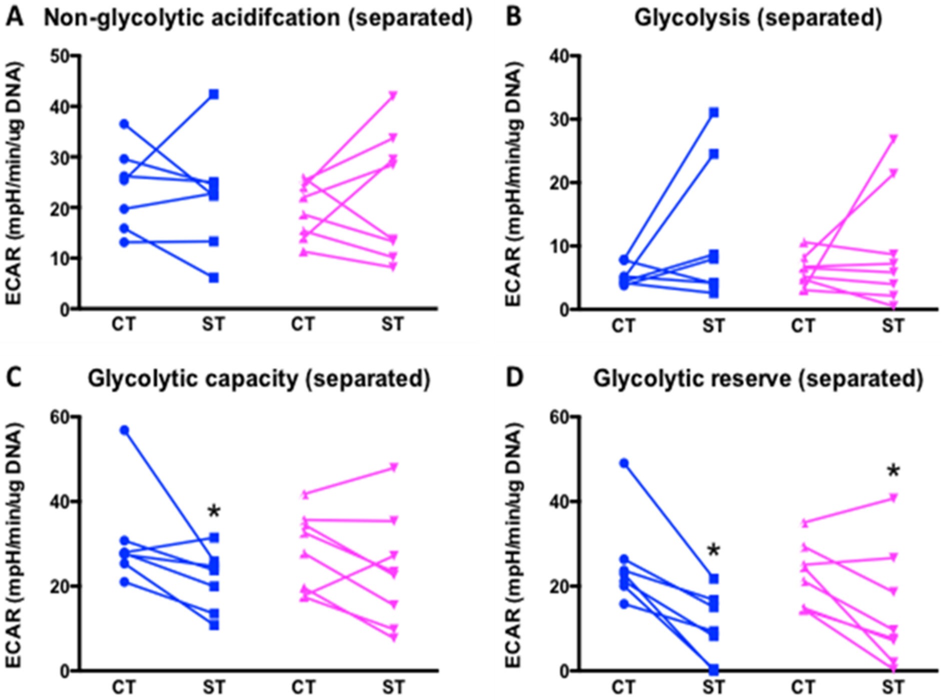

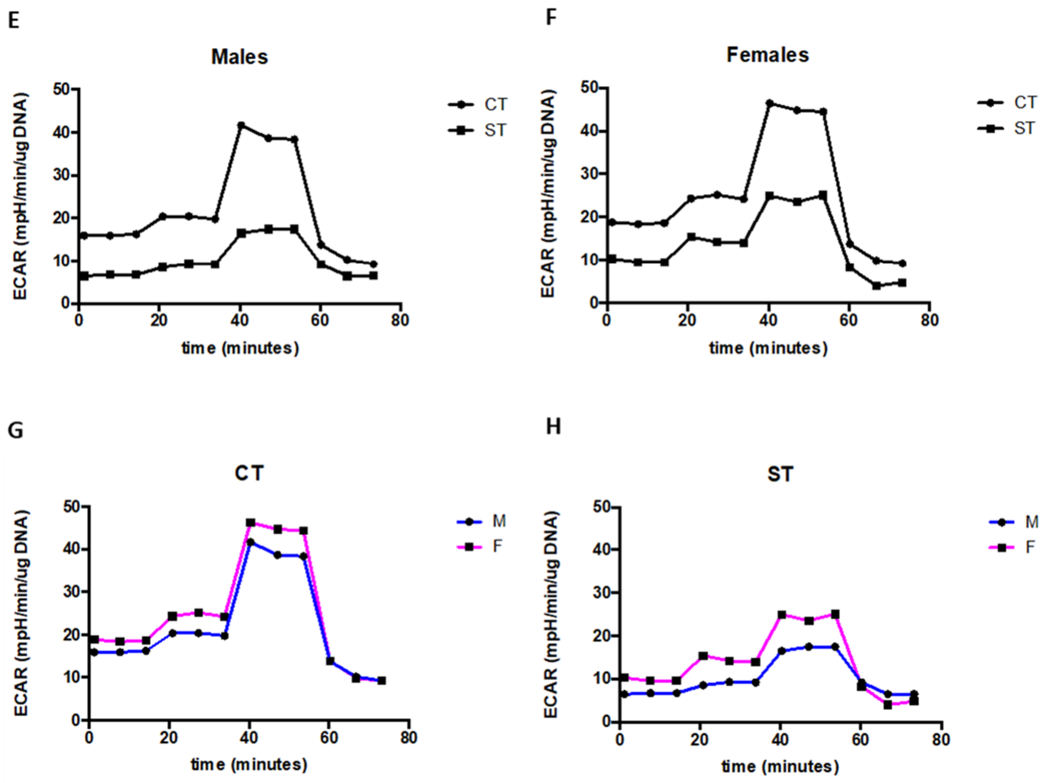
E
Males
ECAR (mpH/min/ug DNA)
time (minutes)
CT
ST
F
Females
ECAR (mpH/min/ug DNA)
time (minutes)
CT
ST
G
CT
ECAR (mpH/min/ug DNA)
time (minutes)
M
F
H
ST
ECAR (mpH/min/ug DNA)
time (minutes)
M
F

Supplemental Figure S3. Mitochondrial function in male and female CT vs. ST

A) Basal respiration, B) ATP-coupled respiration, C) Maximal respiration, D) Spare capacity, E) Non-mitochondrial respiration and F) Proton leak, separated by fetal sex. Male (blue, n=8) and female (pink, n=8), G-J) Data plotted as individual values of paired CT and ST from representative male and female samples. *P<0.05, **P<0.01, P<0.001 (Wilcoxon test CT vs ST).

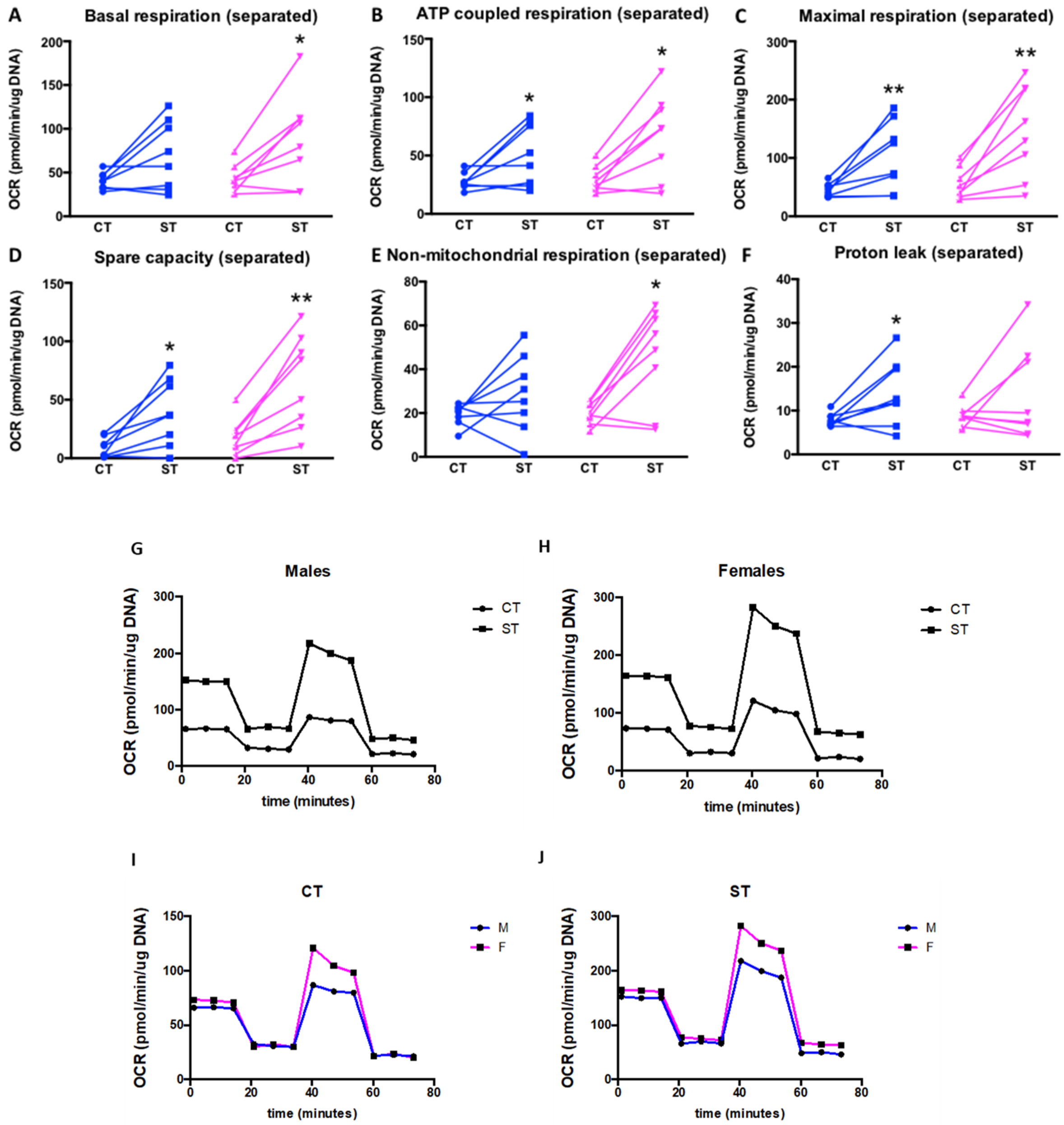

Supplemental Figure S4. Effect of differentiation on mitochondrial mass and function in male and female CT vs ST

A) Mitochondrial mass, B) Citrate synthase protein, C) VDAC protein, D) Citrate synthase activity. Data plotted as individual values of paired CT and ST from the same sample. Data is separated by fetal sex. Male (blue, n=4) and female (pink, n=4). *p<0.05, **p<0.01, (paired t test, CT vs ST within same fetal sex group, and #p<0.05, (Mann-Whitney test, male vs female).

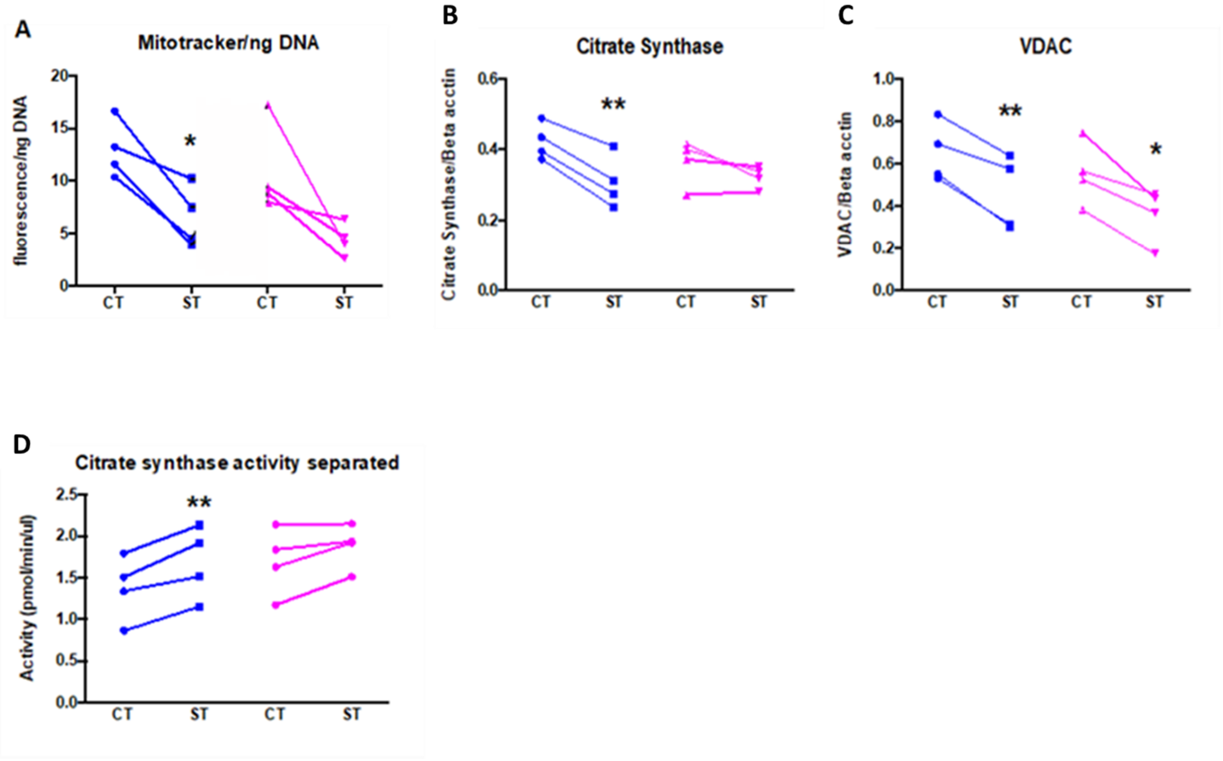

Supplemental Figure S5. Mitochondrial protein expression in male and female CT vs ST

A-K) Data plotted as individual values of paired CT and ST from the same sample. Male (blue, n=4) and female (pink, n=4). Data presented as individual symbols and lines. *p<0.05, **p<0.01, ***p<0.001, (paired t test CT vs ST).

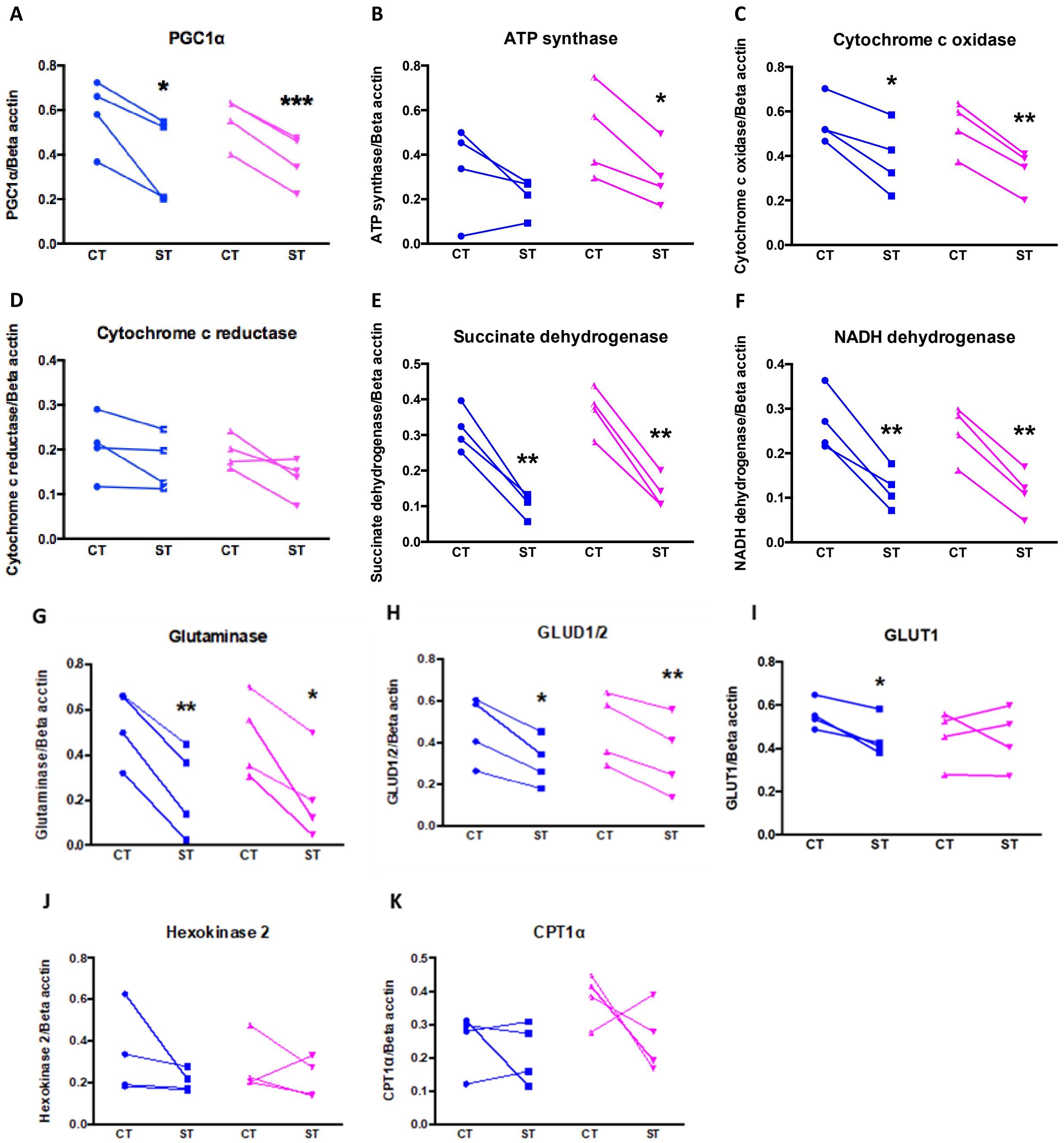

Supplement: Supplementary file 1 [file ijms-22-10875-s001.zip › ijms-1387029-supplementary.pdf]
